# Risk Factors for Transmission of HIV in a Hospital Environment of Yaoundé, Cameroon

**DOI:** 10.3390/ijerph7052085

**Published:** 2010-05-04

**Authors:** Dora Mbanya, Jerome Ateudjieu, Claude Tayou Tagny, Sylvie Moudourou, Marcel Monny Lobe, Lazare Kaptue

**Affiliations:** 1 Faculty of Medicine & Biomedical Sciences, University of Yaoundé I, Cameroon; E-Mails: atdjerom@yahoo.fr (J.A.); tayouclaude@yahoo.fr (C.T.T.); monlobmarc@yahoo.fr (M.M.L.); 2 Centre Hospitalier et Universitaire, Yaoundé, Cameroon; E-Mail: moudsyl@yahoo.fr; 3 Hôpital Central, Yaoundé, Cameroon; 4 Université des Montagnes, Bangangté, Cameroon; E-Mail: prkaptue@hotmail.com

**Keywords:** HIV transmission, health personnel, safety measure

## Abstract

Risk factors for HIV transmission within a hospital setting were assessed using pre-structured questionnaires and observations. Of 409 respondents, 66.3% corresponded to the nursing staff, 14.4% doctors and 8.3% laboratory staff. The irregular use of gloves and other protective clothing for risky tasks, and recapping of needles after use were some of the risk factors identified, especially amongst nurses. Preventive measures were not always implemented by health personnel. More emphasis should be placed not only on diffusing universal precautions and recommendations for hospital staff safety, but accompanying measures for monitoring and evaluation of implementation of these standards are also indispensable.

## 1. Introduction

Nearly three decades after its discovery, HIV/AIDS continues to affect people of all categories worldwide. From four AIDS cases described in 1981, an estimated thirty three million people now live with HIV infection worldwide today, with approximately 70% of them harboured in Sub-Saharan Africa (SSA) [1]. Thus, there is invariably a high prevalence of HIV infection among hospital admissions, along with a high proportion of patients who are probably admitted because of conditions related to their HIV infection or for full blown AIDS. Indeed, very high hospital bed occupancy rates by HIV-infected persons have been reported in some settings of this region, ranging from 20–72% occupancy [2–4]. Hence, inevitably the health care personnel (HCP) in these settings is more exposed to the risk of workplace transmission, compared to other workplaces.

Irrespective of the workplace, employees are often exposed to various types of workplace risks, some of which may be fatal. With the persistently high numbers of people living with HIV and AIDS, its transmission at the workplace cannot be underestimated. In industry, chemicals constitute some of the workplace tools that are corrosive and may irritate and break the skin, with subsequent exposure to higher risk of transmission of infectious pathogens. In other institutions exposure to biological hazards including bacteria, viruses and fungi is the norm, and in yet others, exposure to physical hazards such cuts and injuries are predominant. Sometimes psychological hazards occur from workplace stresses and pressures with consequential lack of concentration and focus on safety measures at the workplace, potentially leading to exposure to health hazards. Thus there is a close link between the working environment and HIV/AIDS. This is more so in the hospital setting, where the risks of contracting HIV infection are higher both for the health care personnel and the patient.

High HIV prevalence among HCP has been reported in some health settings, including a prevalence of 11.5% reported in South Africa [5]. In Cameroon, there is paucity of data on HIV infection among health care workers. Nevertheless, in one study, Polla’a reported an HIV-1 prevalence of 13.9% in a semi-urban health institution of Cameroon and 9.1% among HCP in Yaoundé. In his findings, laboratory staff and nursing aids were predominantly affected in the semi-urban setting, while doctors and brevet nurses were most affected in the urban areas [6].

In a multicentre study in Italy on the risk of occupational human immunodeficiency virus infection in health care workers Ippolito reported 1,592 HIV exposures in 1,534 HCP [7]. In another survey in which needle-stick/sharps injuries and HIV exposure were examined among health care workers in the USA, Henry and Campbell concluded that the number of U.S. hospital workers sustaining these injuries with potential exposure to HIV was considerable [8]. From the early phase of the HIV/AIDS epidemic, some reports already disclosed the risk of not only HIV infection among HCP, but also of others including hepatitis viruses, herpes simplex virus type 2 and cytomegalovirus [9].

According to the International Labour Organization (ILO) [10], the workplace should be where health is protected and accidents and diseases are prevented. This refers to occupational safety and health, which involves the prevention of any detriment to the safety and health of all employees that may arise from exposure to harmful conditions and practices in the work environment. Thus, the work environment should be safe for all.

Although several institutions have introduced HIV/AIDS prevention programmes in their settings, sometimes these have not necessarily been comprehensive, nor have they been strictly implemented. Data from the developed countries suggests very low occupational risk for HCP acquiring HIV. Studies in America and European countries evaluated this risk at 0.34% in America and at 0.37% in Europe, with a 0.001% risk when exposure was through the mucous membranes [11–14]. This is not necessarily true in resource-limited settings where HCP do not always receive adequate training and where the relevant logistics for implementation to protect them against these risks may not always be available. For example, in one study in Nigeria, it was noted that only 15.4% of HCP wore gloves regularly for nursing care [15], while Ngoma reported that 71.42% of HCP in three hospitals of Cameroon always recapped needles after use [16]. These factors would contribute towards occupational risks for HIV transmission. The current guidelines for workplace safety and in particular for post exposure prophylaxis (PEP), insist on HCP taking preliminary precautions to minimize the risk of transmission through the given exposure (washing abundantly with water and others). The HCP is also required to report any accidental exposures to blood of body fluids through cuts, needle prick injuries and direct contacts of these fluids with the mucous membranes and the skin to the appropriately designated persons within each institution.

Thus, the present study was carried out to assess risk factors for and preventive measures against HIV transmission within a hospital setting of Yaoundé (Cameroon) with the aim of identifying and proposing appropriate intervention strategies to curb these risks.

## 2. Experimental Section

### 2.1. Context

This was a hospital-based descriptive study in a major hospital of Yaoundé with a bed capacity of about 500; a patient load of 1,000–1,200 is seen each week and it has a relatively high number of staff, compared to other institutions of the city (in Cameroon the nurse density is estimated to be 1.6 per 1,000 while the doctor density is 0.19 per 1,000). Furthermore, in this institution is more accessible both geographically (centrally located within the city) and financially (lower consultation and hospital admission fees). Thus, it tends to cater for the health needs of all levels of the society.

At the time of the study the services of the hospital included Accident & Emergency (A&E), Surgery and its sub-specialties as well as three operating theatres, Obstetrics-Gynaecology, Paediatrics, Internal Medicine, Laboratory, the Mortuary, Hygiene and Maintenance Unit. There were 681 staff consisting of 100 physicians, 443 nursing staff, 45 laboratory technicians and the rest (93) were administrative, maintenance, hygiene and morgue staff. There was also a hospital pharmacy that dispensed available drugs to patients, according to their prescriptions.

Within the hospital, there were laboratory services for all HIV testing, including exposed staff. Confidentiality was generally maintained for all HIV testing, and for staff in particular, coded names were used to protect their identity. Antiretroviral drugs (ARV) for post exposure prophylaxis (PEP) were available to the staff, free of charge, and obtainable from the hospital pharmacy upon presentation of a prescription, signed by designated physicians within the hospital. All accidental exposures to HIV were expected to be recorded in registers within the services, and reported to a designated nurse staff, who kept all the statistics on exposures within the hospital. Furthermore, the designated physicians were available in the hospital during service hours, and could be called in outside work hours immediately following exposure. They assessed the risk for HIV transmission following exposure, and prescribed appropriate ARV for PEP. The Hospital Pharmacy which dispensed all medication operated 24 hours a day; thus PEP could be accessed as required. No measures were provided for vaccinating HCP against hepatitis B within the institution. The nursing staff was responsible for all nursing care activities including placing and removing of infusions. The laboratory technicians in this institution were responsible for phlebotomy in many cases.

### 2.2. Staff Questionnaire

After obtaining permission from the relevant authorities of the institution (director and heads of different services of the hospital), a list of all the personnel was acquired from each service, including their duty rotation schedules. Several sensitization sessions were held with all personnel within each service, to explain the details of the study. All staff involved with risk activities was included in the study. Appointment days were taken for each service during which a convenience sampling method was adopted, receiving all consenting staff that had spent a minimum of 12 months in the institution, without any discrimination.

Pre-structured, previously-piloted questionnaires were self-administered by all consenting staff supervised by members of the research team, consisting of physicians and medical students, trained to administer the interviews. Each team administering the questionnaire in any given service was constituted of at least a physician and a medical student. The questionnaires had been designed to obtain personal information from the staff (age, sex, category and duration of service, refresher courses attended within the last 1–3 years), as well as general information on the number of staff per service; the available logistics and facilities for HIV prevention including recommended international guidelines. In addition, questions on the preventive measures and attitudes adopted by the health care personnel were included. The questionnaire also contained specific questions on the types and numbers of accidental exposures to blood and body fluids encountered within the last 12 months, the frequencies and the circumstances surrounding them; their official reporting, and the reasons for not reporting. Other questions pertained to various attitudes and practices including when and how often hands were washed, the use of antiseptic solutions, decontamination of soiled material and instrument, the use of protective clothing (white coats, gloves, masks, goggles, rubber boots *etc.*) especially during highly risky acts. Some questions examined their practices in nursing such as locating needles with the fingers, bimanual recapping of needles and scalpels after use, and the disposal of the used needles and sharps. Questions were also asked to find out attitudes and practices towards blood and body fluids that splashed on working surfaces as well as questions on visibility for carrying out certain acts such as phlebotomy.

In the study, all activities that exposed the personnel to blood or other body fluids contaminated with blood were considered as risky acts, while needle-prick injuries, cuts and direct contact with mucous membranes (eyes, mouth *etc.*) or wounds were considered as accidents with exposure to blood and/or body fluids.

### 2.3. Observations in Workplace

An observational grill was used to collect information including the detergents available for cleaning and disinfecting; protective and barrier clothing available and used; hand-washing practices of the personnel, sterilization of instruments as well as on waste disposal. The lighting in different settings was also noted for dullness or brightness, and the records on accidental exposures were also assessed. These direct observational visits were usually impromptu by members of the research team, and were made to the various services of the institution during the six months study period. The findings were noted for analysis.

### 2.4. Data Analyses

All data was masked and entered into a computer programme (MS Excel version 2003) and analyses was carried out by a statistician. Means and standard deviations as well as percentages were used to present the findings.

## 3. Results and Discussion

Of 681 personnel, there were 409 respondents, for a 60.1% response rate. The mean age of the respondents was 38 ± 5 years (range 26–55 years) and 65.3% were females compared to 34.7% males. Their mean duration of service in that institution was 13 ± 6 years (range 1–30 years), thus generally experienced enough to provide relevant and reliable information for the study. With the response rate of about 60% in this study, and in some cases with not all the respondents answering all the questions, there was a variability occasionally observed in the denominators reported in these results.

### 3.1. Staff Repartition by Category and by Service

The staff from Surgery and Paediatrics had the highest numbers of participants in the study (39.3% and 15.4 % respectively). Of the 409 respondents, the majority (66.3%) corresponded to the nursing staff; 14.4% were doctors, 8.3% laboratory staff and the rest (11%) consisted of orderlies and morgue attendants, as well as administrative staff. Table 1 indicates that laboratory technicians and nurses were relatively the largest respondents of the study (75.5% and 61.2% respectively) and the orderlies and morgue attendants were least represented (48.4%). Of 271 respondent nursing staff, 49.4% were from Surgery. There were 59 doctors who participated in the study of which 20 were from Surgery alone (33.9%), with at least 75% of them being surgeons of various categories (general surgeons, urologists, neuro-surgeons, orthopedic and abdominal surgeons, ENT and pediatric surgeons. About 16.9% of all doctors in the study were from the Obstetrics/Gynaecology Service.

### 3.2. Training & Refresher Courses

Concerning knowledge acquisition and updating, some staff of the different services had attended related seminars and workshops on safety within the hospital. The majority (73.1%) had already had at least in-house discussions and instructions on safety within the hospital including lectures within different services organized by trained staff within the said services. In one instance 5 members of staff (1.2% of respondents) had been designated from various services to attend a five-day workshop in another institution of the city on “Safety and hazards within the hospital”. The trained staff was expected to train their colleagues upon return to their various institutions. Other training courses mentioned by some respondents included a half-day lecture on “Accidents and Exposures to Blood and Body Fluids” (12.7% of respondents); a half-day lecture on “Collecting and Transporting Blood Samples and Body Fluids” which was predominantly attended by laboratory staff (61.7% of laboratory staff) and a three-day course on “Quality Assurance within the Hospital” (2.4% of the respondents). Another one-day course which was more recent was on “Hospital Hygiene and the Prevention of Nosocomial Infections”.

### 3.3. Reports on Available Preventive Material & Equipment

Safety guidelines were displayed on walls and notice boards throughout the hospital and standard operating procedures were reported as available in some services. Facilities and logistics for sterilization were reported as available in all services, but were not always functional, while soap, antiseptic solutions and detergents were constantly available in only three services; the rest had frequently interrupted stocks of these detergents (Table 2).

#### 3.3.1. Personal protective clothing/equipment

Gloves (latex) were mostly available, although stock-outs were sometimes reported or noticed (see Table 2). Masks and impermeable aprons were mostly seen in surgical theatres while protective goggles and face shields were virtually non-existent. It was also observed that gloves made of nitrile or butyl rubber were rare.

#### 3.3.2. Waste disposal

All services had waste *bin buckets with covers* and plastic bags for containing rubbish. Plastic containers for sharps were reported to be always available. An incinerator was present in the hospital.

### 3.4. Reported Record Keeping

Registers for exposures and/or accidents occurring to staff or patients were reported in the questionnaires as not available in all the services except in two.

### 3.5. Risk practices & Attitudes Reported

#### 3.5.1. Overall preventive activities

Of 350 the respondents to the question, 45.4% washed their hands before each activity, 70.9% washed hands after each activity and 52.7% regularly used gloves for risky acts (cleaning soiled instruments, blood spilled on surfaces). Of 344 respondents, there was a 100% glove usage for all surgical procedures, these by Doctors. About 84.9% of the respondents always cleaned, disinfected and sterilized used instruments for next use and 83.1% always used disposable surgical blades for various procedures, especially the doctors (100%). Over 70% of respondents cleaned splashes onto working surfaces with disinfectants for over 10 minutes, mainly the laboratory technicians (72.4%) and least of all, the orderlies (4.3%).

#### 3.5.2. Risky acts

A total of 75.7% of 342 respondents to the question reported that they usually recapped needles after use. More than 90% of these were nurses. Another 34.7% washed and/or disinfected used gloves for reuse, predominantly laboratory staff; 16.9% sterilized and reused disposable surgical blades and 15.1% cleaned and disinfected instruments for reuse.

Of 47 respondents to a question on the use of scalpels during surgery six personnel in Surgery (12.8% of respondents) and one in Obstetrics/Gynaecology (2.1% of the respondents), all of them physicians, admitted to using scalpels without mounting them on the handles, and some had actually been injured from that. Similarly, concerning the use of forceps for securing needles for suturing wounds, three of 48 respondents (6.1%) did not use them at all, while five (10.2%) used them irregularly. The majority of these were doctors and a few nurses from A&E and the theatre. There were 21.9% Laboratory staff who admitted to occasionally using pipettes by mouth.

#### 3.5.3. Exposures and accidents

Within the previous 12 months to the study, at least one needle-prick injury had occurred in 196 of 323 respondents to that question (60.7%), this mainly during recapping. There were 157 of these (80.1%) who reported using bimanual recapping methods. Although no accidents seemed connected to the locating of needles with the fingers, there were 65 respondents who reported the habit. Of 359 respondents to the question 44.8% (161) declared haven had direct contact with body fluids at least once, mainly on the hands, the eyes and the mouth. The nurses and doctors were most affected by the various accidents (Table 3).

As shown in Table 4, most exposures and accidents occurred during bimanual needle recapping and removal of needles from the veins, implying that these were hollow needles and in some cases they were large-bore needles. A high percentage of HCP were exposed to injury through the removal of needles from the vein (45.9%), this being a high risk exposure. Up to 8% exposures occurred from the overfilled sharps containers. Though not a high risk exposure, needles with large bore containing blood and freshly out of the blood vessels could constitute very risky exposures. Interestingly very few of the exposed personnel had made official reports of these exposures, mainly for fear of being screened for HIV prior to prophylaxis ARV (90%). Table 5 indicates the major circumstances that exposed HCP to body fluids including deliveries, the cleaning of instruments and surfaces as well as intubations during endoscopy).

#### 3.5.4. Reported measures taken, following risk exposures

Among 196 respondents with injury, 57% squeezed and bled the wound; 81.1% washed the spot with soapy water and antiseptic solutions; 12.4% washed the injury with ordinary tap water and about 6.6% had done absolutely nothing. Of 161 exposed to splashes of body fluids, 61 (37.8%) washed the exposed parts with a lot of water while the rest took no particular precautions.

#### 3.5.5. Results from direct observations

Discrepancies were noted between responses in the questionnaires and the actual practices observed. For example over-filled sharp containers were seen to carelessly stay around on about five occasions in laboratory services and in A&E, and twice, sharps were seen in ordinary waste bins. On probing at the time, some explanations included unavailability of appropriate containers and sometimes the few overworked staff could not find a moment to attend to these. In all the services of the hospital, used mineral water bottles and sometimes bottles of more resistant materials were observed to serve as sharp containers. A nurse and a laboratory technician were noted not to cover their wounds with sticky plaster before putting on gloves, let alone coming to work without protecting cuts.

Work surfaces with splashes of blood or body fluid were not always disinfected with antiseptic for the specified durations as recommended before cleaning. Sometimes orderlies were observed to clean floors and other messy places, and in some instances transported rubbish in plastic bags, without any gloves. No reinforced gloves (made of nitrile or butyl rubber) were ever noticed, even for very risky tasks. At least 50% of doctors had packs of gloves in their offices, for their personal use. A few laboratory technicians (about 25%) also had a few gloves in their pockets to use and reuse for risky acts.

In some wards and the laboratory, the lighting in the work areas was not always very bright, and some had to displace patients in order to prick their veins. In the laboratory, staff was observed to occasionally go outside the building in order to read certain visual results clearly.

Concerning registers for reporting accidents, only two were seen and one just consisted in mentioning incidents besides the names of the concerned, in a general consultation register.

### 3.6. Discussions

In Cameroon the national HIV prevalence has been reported as 5.5% of approximately 16 million people [17]. Polla’a reported a prevalence of HIV infection of 13.9% and 9.1% respectively among HCP in a semi-urban and urban health institution of Cameroon [6]. These, coupled with the findings of this study all demonstrate the continuous need for assessing not only the transmission of knowledge and the availability of infrastructure and logistics, but also of staff performance in any institution, as this contributes significantly towards abiding by safety rules and decreased exposure to risks.

There were 60.7% cases of exposure to blood and body fluids through needlestick injuries *etc.* reported in this study, mainly in nurses and doctors. Of all the respondents 49.4% and 33.9% were respectively nurses and doctors from Surgery, these being amongst the high risk group for exposure. Up to 45.9% of the respondents had risk exposures through the removal of needles from the vein. Accidents related to the removal of needles from the vein in themselves constitute a high risk activity for pathogen transmission for HCP and should be curbed. Such high exposure rates have also been reported in other African studies. For example, in a multicentre study in West Africa (Cote d’Ivoire, Mali and Senegal), Tarantola and his collaborators reported 45.7% exposure of HCP to blood and body fluids [18]. This further emphasizes the need for the continuous education of all health care workers on the Universal Precautions regarding the safety and the handling of blood, body fluids and contaminated instruments. Ippolito and his collaborators reported needlestick injuries to be the most common source of exposure in their study, and observed that most occurred in nurses [7].

Although several HCP had undertaken various short courses on safety within the hospital and laboratories, these were not necessarily implemented. For example, universal regulations require that hand-washing be practiced regularly before and after different acts in the hospital, even without soap. It was noted in this study that only 45.4% and 70.9% of respondents washed hands before and after various acts. Admittedly, there are sometimes interruptions of water supply, but this was not the main reason for this practice. HCP also need to play their part in complying with the available universal precautions.

The use of barrier clothing was not always respected, mainly because of availability. All staff had white coats at all times, but even latex gloves were frequently out of stock and aprons, masks and goggles were restrictively accessible. In some instances gloves made of nitrile or butyl rubber are recommended, to avoid skin contact, but even latex gloves which may not provide adequate protection in those instances were sometimes unavailable to HCP. Similar findings have been reported in a study on the transmission of tuberculosis and HIV in a hospital environment in Cameroon [19]. With other priorities within the hospital, and with little available resources, some services are obliged to prioritize their needs. This would explain why gloves could be used 100% for surgical procedures, and not necessarily used in other services. This would also explain various risky activities such as the washing and reusing of used gloves and syringes, in an era when disposable material is recommended and when the doubling of gloves is encouraged in some circumstances. The stakes are high. In Tanzania, Hoelscher and his collaborators reported that of 1,219 syringes, sterilized and ready for use, 3 of them (0.25%) had HIV and 32.8% had other bacterial germs [20]. This finding further stresses the importance of the single use of disposable equipment and material at all times, and as much as possible.

It was noted that sometimes the containers for sharps were over-full, and not discarded promptly, further exposing the staff to risks. There were various reasons provided for this, mainly inconsistent availability of these containers. In some instances, the small numbers of staff in a service reported being so overworked that they seemed to find no minute to promptly replace the full containers. Furthermore, and from observations, the designated containers were not always used, as sharps were seen in ordinary bin bags. In his analysis, Bougataya [21] observed that most HCP dumped sharps in ordinary plastic dustbin bags, from unavailability of appropriate containers.

The recapping of needles after use was a popular finding among HCP. Bimanual recapping was usually of needles used for both intramuscular and intravenous injections and phlebotomy, implying that these were hollow needles, these being higher risk for pathogen transmission compared to full needles. Where the hollowed needles were large-bore needles, or where the needles had come directly from out of the blood vessels, the risk was higher. Nevertheless, this study did not decipher the numbers for each category of needle. A high percentage of HCP were exposed to injury through the removal of needles from the vein (45.9%), this being a high risk exposure. Up to 8% exposures occurred from the overfilled sharps containers. Other studies continue to report this finding of needle recapping after use including Bougataya who reported this in 81.6% of HCP in his study [21]. One reason for this persistent risky practice has to do with “old” habits. Concerning the bimanual recapping of used needles, this is discouraged by all universal precautions. And if recapping of needles must be done, it should respect the single-handed recapping method. Furthermore, other risky practices including the locating of needles with fingers reported in this study, is not recommended. In this study, it was also noted that sometimes medical procedures were carried out with minimal lighting, allowing for greater risks of accidents. The placing and removing of drips and transfusion sets was also a major cause of accidents reported in this study, and these procedures require adequate lighting. However, sometimes accidents were reported to occur because several risk objects were being manipulated simultaneously.

Although several respondents had been exposed to injuries and accidents, there were no official reports of the accidents that occurred. Hence during the study period, there was no Post Exposure Prophylaxis administered for HIV or other pathogens. Every case declared their reluctance to get an HIV test performed as part of the recommended procedure for starting ARV, and monitoring for the risk of transmission of infections. Indeed preliminary unpublished data in 2001 from a hospital of Yaoundé reported very low rate of PEP following staff exposure to blood and body fluids, with only 24% cases reporting on time, within 48 hours (Kouanfack C, unpublished communication). Currently, PEP recommended in Cameroon involves the administration of highly active antiretroviral therapy for a month, with surveillance of HIV and other infections at prescribed intervals (one, three and six months) for up to six months. It is not obvious why there is such recalcitrance towards declaring exposures and hence towards PEP, but fear of stigma may be one reason as well as fear of coping with the side effects of ARV. However, the questionnaires in this study did not specifically address these issues. Other studies have also noted the under-reporting of these accidents, with under-reporting rates ranging from 60% to as high as 100% in some cases [21–25]. Concerning precautions taken after accidents, many declared squeezing out the blood and washing the injured spot. The procedure of squeezing out blood from such injury is discouraged, as this may further irritate surrounding tissue and favour transmission.

There were several limitations to this study including the relatively low response rate (60.1%) and the disproportionate representation of staff from the different services in the study. In addition, no data was collected on the non-respondents to allow comparison with the respondents and possible biases within the two groups. Furthermore, some staff categories were relatively under-represented including orderlies and morgue attendants, these also being an at-risk group for disease transmission at the workplace. Ideally all staff was the target, but not all could be available as some were inevitably absent when their services were visited, including those on maternity, sick or annual leave. Others were too busy to spare time for the interviews. The study did not focus on assessing knowledge of the respondents of specific aspects of HIV/AIDS, nor did it specifically examine stigma, which may itself constitute a risk factor. However, an earlier study in a rural hospital setting of Cameroon about 70% of the nurses and laboratory staff scored highly in their knowledge on HIV, compared to about 50% in attitude. In addition, this study reported that fear of being infected during patient care influence stigma and discrimination in patient care [26]. In Kenya, women were reported to avoid delivery in health facilities for fear of stigma and discrimination [27]. Such believes and attitudes by HCP would logically influence the health care provider’s own willingness to be identified as infected by the virus. Indeed one study has linked stigma and fear as a main reason for the migration of nurses in African countries [28].

It may be concluded, depending on the risk activity analyzed and the preventive measures taken, and depending on the exposures reported here, that virtually all categories of the HCP showed insufficiencies, suggesting that intervention strategies are needed that target all categories of these HCP.

## 4. Conclusions

Like most developing countries, resources are scarce and stock-outs are frequent in Cameroon. Hence, despite acquisition of knowledge through refresher courses, some of the personnel practices and attitudes remain unchanged and they do not conform to available universal precautions. This is aggravated by the difficulty in abandoning old habits, especially in the long-serving staff. The frequent shortages of protective materials increase risks for the transmission of infectious pathogens, suggesting a great need for resource mobilization as well as prioritization of resources to provide protection for those seeking to give care to others. Furthermore, the reporting of accidents and exposures to the appropriate hospital departments should be encouraged, in order to ensure proper follow-up and management of the affected HCP. It is the responsibility of any institution (employer) to guarantee the safety of its personnel and should therefore provide single-use consumable items such as needles, syringes, gloves, and in adequate quantities.

This study did not evaluate every aspect of occupational safety and health in a hospital setting, but confirms the need, not only for capacity building and human resource development as the cornerstone to success in any institution, but also for a coordinated advisory or governing body in the hospital settings (identify and train appropriate staff) designated to monitor and evaluate the implementation of internationally approved guidelines.

Thus, to the institution, we recommend that personnel protective equipment and material be made available and allocated at all times, prioritized according to service needs to ensure staff safety, and that basic needs such as disinfectants and detergents be constantly available. Precautions should be taken to ensure that needles with needlestick protective features are obtained. Specific staff should be identified and trained to monitor and evaluate staff safety within the hospital, including establishing a surveillance system that collects all information on exposures to risks and their eventual outcomes.

To the HCP, they have the responsibility of respecting recommended norms, using resources provided and caring about their own safety. They should be responsible enough to declare all exposures to enable the institutions take appropriate action. There is a need for the development of performance indicators and standards for the compliance levels of health care personnel to international norms within the hospitals.

## Figures and Tables

**Table 1.** General distribution of the respondents by professional category.

**Staff Category**	**Total numbers in the hospital**	**Total numbers of respondents**	**% Respondents**

*Nurses	443	**271**	**61.2**
*Doctors	100	**59**	**59.0**
*Lab technicians	45	**34**	**75.5**
Orderlies, morgue attendants & others	93	**45**	**48.4**

**Total**	**681**	**409**	**60.1**

* Includes all grades and categories.

**Table 2.** Availability of material and equipment by service.

**Service**	**Material/Equipment**										
	*Safety notices	**Soap**	**Anti-septic solutions**	**Detergent**	**Masks**	**Goggles**	**Impermeable Aprons**	**Rubber boots**	**Gloves**	**Dry heat sterilizer**	**Autoclave**

ENT		IRR	IRR	P	A	A	A	A	A	P	A
Surgical Units	P	IRR	IRR		A	A	A	A	P	P	A
ICU		IRR	IRR		A	A	A	A	IRR	P	A
Stomatology		IRR	IRR		A	A	P	A	IRR	P	A
Theaters		P	P	P	P	IRR	P	A	P	P	P
A & E	P	IRR	IRR	IRR	A	A	A	A	IRR	P	A
Paediatrics	P	IRR	IRR	IRR	A	A	A	A	IRR	P	A
Internal Medicine		IRR	IRR	P	A	A	A	A	IRR	OOO	A
Obs/Gyn	P	IRR	P	P	A	A	A	A	P	P	A
Laboratory	P	IRR	IRR	P	A	A	A	A	IRR	P	A
Hygiene &Maintenance Unit	P	P	P	P	A	A	A	P	P	P	A

ENT = Ear/Nose/trachea;

ICU = Intensive Care unit;

P = Present constantly;

IRR = Irregularly present;

A = Absent;

OOO = Out of order;

* = As observed by researchers.

**Table 3.** Exposure and Accidents rate by job category.

**Staff category**	**Exposure to needle prick injuries**			**Exposure through contact with body fluids**		

	**Total respondents**	**No. Exposed to needle pricks (%)**	**% on overall No. Exposed (n = 196)**	**Total respondents**	**No. exposed to body fluids (%)**	**% on overall No. Exposed (n = 161)**

Doctors	48	37 (77.1)	18.9	36	17 (42.2)	10.6
Nurses	216	132 (61.1)	67.4	253	118 (46.6)	73.3
Lab technicians	32	19 (59.4)	9.7	32	17 (53.1)	10.6
Orderlies, Morgue attendants	27	8 (29.6)	4.1	26	7 (26.9)	4.4

**Table 4.** Circumstances for risk exposures to needle prick injuries.

Circumstance*	**Total numbers exposed (n = 196)**	
	**n**	**Percentage**

Bimanual needle recapping	131	66.8
Disconnecting needle from drip sets, transfusion sets…	34	17.3
Needle left carelessly	32	16.3
Overfilled sharps’ container	16	8.2
Faulty manoeuvre	26	13.3
During cleaning of instruments	30	15.3
During injection administration	13	6.6
During needle removal from vein**	90	45.9
Surgical incision**	22	11.2

* Some had > 1 circumstance

** High risk exposures.

**Table 5.** Circumstances for risk exposures through contact with fluids.

Circumstance*	**Total number exposed (n = 161)**	
	**n**	**Percentage**

Venous sample collection	11	6.8
Lumbar tap	20	12.4
Intubations for endoscopy	26	16.2
Minor surgery	10	6.2
Various nursing procedures on the wards	24	14.9
Cleaning materials and surfaces	27	16.9
Delivery	31	19.3
Major surgery	23	14.3

* Some had>1 circumstance.
